# DMN-YOLO: A Lightweight Small-Object Detector for Multi-Species Animal Detection in UAV Grassland Imagery

**DOI:** 10.3390/ani16111643

**Published:** 2026-05-27

**Authors:** Qian Huang, Jun Yang, Mengqi Yang, Dan Jiang, Tan Wang

**Affiliations:** 1 School of Artificial Intelligence, Anhui Agricultural University, Hefei 230036, China; 2 Key Laboratory of Agricultural Sensors, Ministry of Agriculture and Rural Affairs, Anhui Agricultural University, Hefei 230036, China

**Keywords:** unmanned aerial vehicle, grassland monitoring, small object detection, lightweight model

## Abstract

To meet the requirements of accurate multi-class animal detection and model lightweighting in UAV-based grazing monitoring, this study presents DMN-YOLO, an efficient detector built upon YOLO11n. In particular, a lightweight downsampling module, DSDown, is introduced to alleviate the loss of detailed features of tiny targets during downsampling under complex grassland backgrounds, thereby improving the preservation of edge, texture, and local structural information. Meanwhile, a MACFPN multi-scale feature fusion structure is designed to handle large scale variations and feature confusion among multiple animal targets, enhancing cross-scale feature interaction and background suppression for better small-target representation. In addition, NWDR Loss combines CIoU geometric constraints, normalized Wasserstein distance, and an adaptive weighting strategy to improve overall stability and localization accuracy of small-target bounding box regression. Results indicate that DMN-YOLO attains 93.6% precision, 89.9% recall, and 95.8% mAP@0.5 on the UAV animal detection dataset. Compared with YOLO11n, it reduces the parameter count by 35.7% while lowering the model size by 29.3%. These results show that DMN-YOLO effectively reduces model complexity while maintaining strong detection performance, demonstrating good potential for practical field deployment.

## Simple Summary

Monitoring animals in open grasslands is important for grazing management, wildlife protection, and ecological conservation, but traditional field surveys are often slow, labor-intensive, and limited by terrain. Drone images provide a more efficient way to observe animals over large areas, but animals often appear very small in aerial images and can be difficult to distinguish from grass, shadows, and other background objects. This study developed a lightweight computer-based method to detect multiple animal species, including cattle, sheep, and wild animals, in drone images of grassland scenes. The method was designed to improve the detection of small animals while keeping the model compact enough for practical use. The results showed that the proposed method correctly identified most animal targets and performed better than the original model while using a smaller model size. This study provides a practical tool for animal monitoring, grazing management, ecological patrols, and intelligent grassland conservation.

## 1. Introduction

Grazing-based grassland livestock production is essential in supplying meat and milk and in safeguarding herders’ livelihoods in many regions [1]. In open rangelands, livestock such as cattle and sheep often share grassland resources with wild herbivores such as kiangs, making grassland carrying capacity and the coordinated management of livestock and wild herbivores an important issue in pastoral-area management [2]. To support grazing management and patrol-based monitoring, it is necessary to continuously acquire and dynamically update information on the spatial distribution and population changes in livestock [3]. Traditional field inspections mainly rely on manual observation and are strongly constrained by terrain and other environmental conditions, resulting in limited coverage and delayed information updating, which makes them insufficient to satisfy the demands of large-scale, routine, and fine-grained grazing management [4].

In recent years, unmanned aerial vehicles (UAVs), with their advantages of flexible mobility and convenient deployment, have provided a more efficient means of information acquisition for animal monitoring in grazing areas [5]. UAV aerial imagery enables more frequent acquisition of information on animal spatial distribution, abundance dynamics, and aggregation patterns, thereby improving the timeliness of monitoring and management. However, animal detection in UAV imagery still faces considerable challenges. Owing to flight altitude and imaging-resolution constraints, animal targets often appear as small-scale or even extremely small-scale objects. At the same time, dense distribution, mutual occlusion, complex backgrounds, and illumination variation may result in missed detections and false detections [6].

With the growing application of deep learning in UAV remote sensing and precision livestock farming, increasing attention has been paid to detection and deployment schemes better suited to grazing scenarios characterized by small, densely distributed, and easily occluded animal targets. Yang et al. proposed an improved YOLOv5s-based forest wildlife detection algorithm by introducing weighted channel concatenation, a Swin Transformer module, and an α-DIoU loss function, achieving an mAP of 89.4% and demonstrating good performance in complex animal detection scenarios [7]. Wang et al. based on YOLOX-nano, constructed an enhanced backbone and a feature pyramid fusion module for grassland grazing livestock detection, achieving a composite detection metric of 86.47% [8]. Cao et al. incorporated efficient channel attention (ECA) into YOLOv5x and combined it with tracking-based counting for dynamic sheep counting in UAV videos, with the detection accuracy on the test set reaching 97.10% [9]. Ye et al. developed an improved YOLOv8n-derived model for animals from UAV imagery, which improved mAP@0.5 by 7.1% over the baseline on their dataset [10]. To address dense livestock distribution, occlusion, and the difficulty of recognizing small targets under complex grassland backgrounds, the study of Fang et al. proposed an enhanced CCS-YOLOv8 framework by incorporating CBAM, CARAFE, and a small-object detection layer, and reported an mAP@0.5 of 84.4% and a F1-score of 83.1%, demonstrating a favorable balance between accuracy and real-time performance [11]. Focusing on the low pixel proportion of targets in aerial counting of high-density sheep flocks, Biggs et al. proposed a sub-window inference method and combined it with random-cropping augmentation for training, achieving a sheep-counting MAE of 3.21 together with a MAPE of 1.27%, which indicates strong applicability in high-density small-target scenarios [12].

Existing UAV-based animal detection studies mostly focus on a single target type, and relatively few have addressed the coordinated detection of livestock such as cattle and sheep together with wild herbivores such as kiangs. Therefore, existing methods are still insufficient for supporting synchronous perception and dynamic monitoring of multiple target categories in complex grazing environments. Compared with single-species detection, multi-species animal detection in grassland scenarios faces more complex technical challenges. On the one hand, animal targets in grassland aerial images are usually small in scale and weak in edge information, and they are easily affected by grass textures, shadows, illumination changes, and background regions with similar appearances. During continuous downsampling, lightweight networks may weaken or even lose the edge, texture, and contour information of small animals, resulting in missed detections and localization errors. On the other hand, cattle, sheep, and wild herbivores differ in scale and imaging viewpoint, and their features may become confused after feature compression, which increases the difficulty of multi-class discrimination. Meanwhile, UAV-based grassland monitoring usually requires real-time deployment on edge devices, imposing higher requirements on model parameter size, computational cost, and inference speed. However, existing improved models often focus on optimizing a single aspect: complex structures may improve detection accuracy but increase deployment cost, whereas excessive lightweighting may sacrifice the detailed features and localization ability of small targets. As a result, it remains difficult to simultaneously achieve small-target feature preservation, multi-class discrimination, and efficient edge-side deployment. Therefore, multi-species small-object detection under complex grassland backgrounds still requires a more targeted lightweight model.

To address the above issues, this study develops DMN-YOLO based on YOLO11n for multi-species small-object detection of livestock such as cattle and sheep, as well as wild herbivores such as kiangs, under complex grassland backgrounds, with consideration of edge-side deployment. The main improvements of this study are as follows:

(1) To meet the lightweight requirements of edge deployment and address the loss of detailed information of small animal targets during downsampling, a lightweight downsampling module, DSDown, is designed. This module reduces computational cost while enhancing the preservation of edge, texture, and local structural information of small targets.

(2) To address the large scale variations and feature confusion among multiple animal categories under complex grassland backgrounds, a MACFPN multi-scale feature fusion structure is constructed. By aligning and integrating features from different levels, this structure enhances cross-scale feature interaction and background suppression, thereby improving the feature representation and category discrimination of multi-species small targets.

(3) To address the problem that localization deviations of small targets can easily lead to reduced detection accuracy, NWDR Loss is proposed. By strengthening the bounding-box regression constraints for small targets, this loss improves small-target localization accuracy and overall detection performance.

## 2. Materials and Methods

### 2.1. Dataset Construction

**Figure 1:**
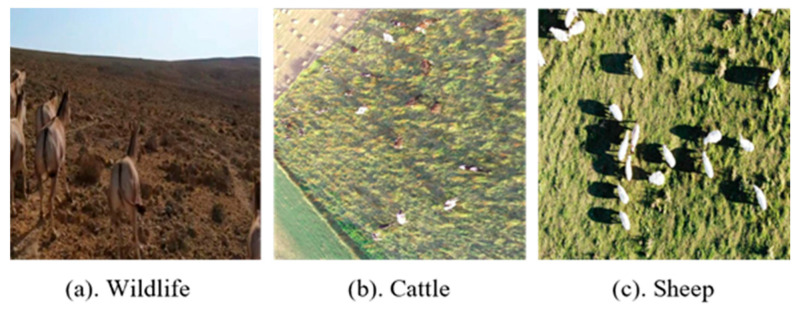
Sample images from the WAID dataset.

The experimental dataset was constructed based on the WAID UAV animal detection dataset [13]. According to the requirements of this study, three target categories, namely wild animals, cattle, and sheep, were selected from the original dataset as the detection objects, with representative samples shown in Figure 1. The final dataset was divided into training, validation, and test sets, containing 4613, 1312, and 702 images, respectively, and all images have a resolution of 640 × 640 pixels. The annotation box counts of the three animal categories in each dataset split are summarized in Table 1.

### 2.2. Method

**Figure 2:**
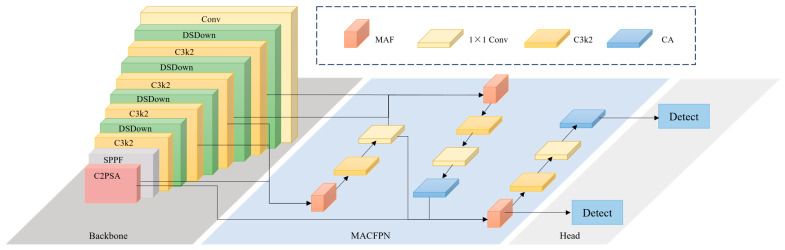
Overall structure of the DMN-YOLO model.

To enhance the detection capability of UAV-based animal monitoring under complex grassland backgrounds, this study proposes DMN-YOLO based on YOLO11n [14]. To address the loss of detailed feature information during downsampling, the DSDown module is designed to better preserve edge and texture details of small targets while maintaining a lightweight architecture. To address feature confusion among multi-class animal targets and insufficient interaction among features at different levels, the MACFPN structure is constructed to strengthen cross-scale feature interaction and suppress background interference. In addition, NWDR Loss is proposed to strengthen the learning of small-object bounding-box prediction, thus improving localization accuracy and training stability. These improvements collaboratively optimize feature representation, multi-scale fusion, and box regression, enabling the proposed network to achieve better small-object detection performance while retaining good deployment efficiency. The overall architecture of the proposed framework is illustrated in Figure 2.

#### 2.2.1. DSDown

**Figure 3:**
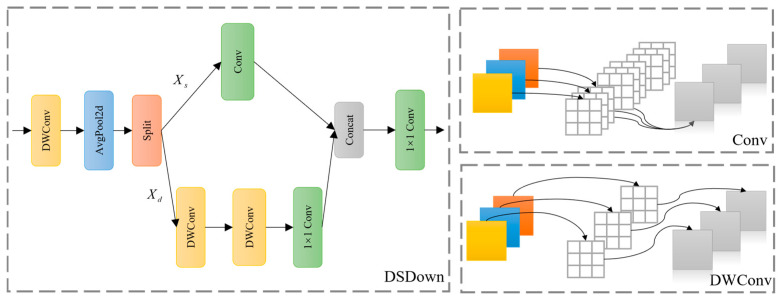
Structure of the DSDown module.

To cope with the challenges in UAV-based grassland monitoring, including dense background textures, illumination variation, small targets, and weak edge responses, a lightweight downsampling module termed Dual-branch Semantic-Detail Downsampling (DSDown) is introduced. As shown in Figure 3, this module aims to preserve target-related structural details while reducing spatial resolution.

Given the input feature map X, a lightweight preprocessing branch is first applied to suppress high-frequency interference caused by grass textures and shadow fluctuations:

(1) $$X_{a}=\mathrm{SiLU}(\mathrm{BN}({\mathrm{DWConv}}_{3\times 3}(X)))$$

where ${\mathrm{DWConv}}_{3\times 3}(\cdot )$, BN(⋅), and SiLU(⋅) denote depthwise convolution [15], batch normalization, and the activation function, respectively. After preprocessing, average pooling is used to aggregate local receptive field information, and the feature map is divided along the channel dimension into a semantic branch and a detail branch:

(2) $$X^{\prime }={\mathrm{AvgPool}}_{2,1}(X_{a}),[X_{s},X_{d}]=\mathrm{Split}(X^{\prime })$$

The semantic branch focuses on high-level discriminative information such as target contours and regional morphology, and performs spatial downsampling through a 3×3 strided convolution:

(3) $$F_{s}={\mathrm{Conv}}_{3\times 3,s=2}(X_{s})$$

The detail branch is designed to preserve edge, texture, and local structural information. It adopts lightweight convolutions to complete downsampling and channel adjustment at a low computational cost:

(4) $$F_{d}={\mathrm{Conv}}_{1\times 1}({\mathrm{DWConv}}_{3\times 3,s=2}({\mathrm{DWConv}}_{3\times 3,s=1}(X_{d})))$$

To improve the flexibility of feature utilization, a gating mechanism is introduced to adaptively assign branch weights according to the global response of the input feature [16]:

(5) $$\left[\alpha ,\beta ]=\mathrm{Softmax}({\mathrm{Conv}}_{1\times 1}(\mathrm{SiLU}({\mathrm{Conv}}_{1\times 1}(\mathrm{GAP}(X_{a}))))\right)$$

where α and β denote the coefficients of the semantic branch and the detail branch, respectively, with α+β=1. The weighted branch features are subsequently merged and fused by a lightweight convolution layer to generate the output feature map:

(6) $$F_{out}={\mathrm{Conv}}_{1\times 1}(\mathrm{Concat}(\alpha F_{s},\beta F_{d}))$$

By combining semantic information with fine-grained detail preservation, DSDown improves the effectiveness of downsampling under complex grassland backgrounds while maintaining a lightweight architecture, thereby providing more compact and discriminative features for subsequent detection.

#### 2.2.2. MACFPN

**Figure 4:**
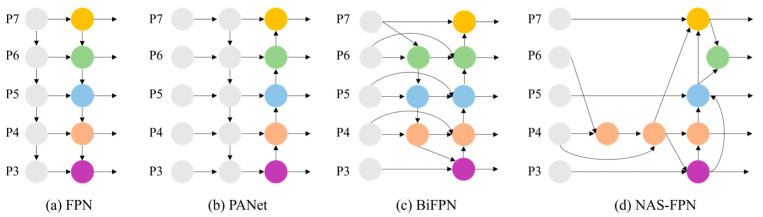
Structural comparison of representative feature pyramid networks.

The neck module is responsible for multi-scale feature propagation and fusion. Common feature fusion structures include FPN, PANet, BiFPN, and NAS-FPN, and their structural differences are shown in Figure 4. Specifically, FPN enhances multi-scale semantic representation through a top-down pathway and lateral connections; PANet further introduces a bottom-up path based on FPN to strengthen feature propagation; BiFPN introduces bidirectional fusion and learnable weights to improve multi-scale fusion efficiency; and NAS-FPN obtains cross-scale fusion topologies through neural architecture search. However, these structures may still be affected by progressive scale transformations during feature fusion, making it difficult for effective fine-grained information of small objects to be sufficiently propagated to subsequent detection layers. For UAV-based grassland small-animal detection, the targets occupy only a small proportion of the image, and detailed localization information is gradually lost after repeated downsampling. Low-level high-resolution features contain more localization and detail information but have weaker semantic representation, whereas high-level low-resolution features contain stronger semantic information, but the spatial details of small objects have already been compressed. To overcome this problem, this study introduces a Multi-Scale Aligned Fusion Feature Pyramid Network (MACFPN) as an enhanced neck structure, allowing high-resolution detail features, intermediate-scale structural features, and low-resolution semantic features to jointly participate in cross-scale fusion, thereby strengthening the transmission and reuse of small-object detail information and deep semantic information in the neck stage.

**Figure 5:**
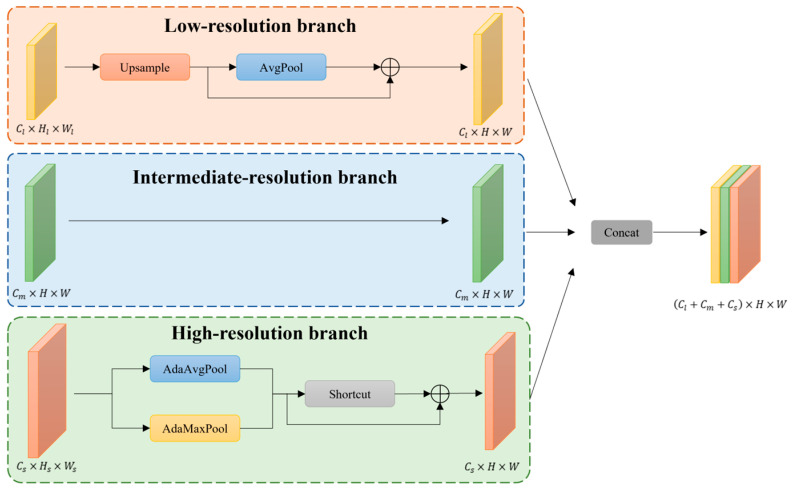
Architecture of the MAF module.

Specifically, this study develops a multi-scale aligned fusion (MAF) module to strengthen feature aggregation across different resolutions, as illustrated in Figure 5. Denote the input features as the high-resolution feature $X_{l}$, the intermediate-resolution feature $X_{m}$, and the low-resolution feature $X_{s}$, where the spatial size of $X_{m}$ is denoted as S. The intermediate-resolution feature is used as the alignment reference.

For the high-resolution branch, which has a higher spatial resolution and contains richer detailed information, adaptive max pooling and adaptive average pooling are jointly adopted for scale alignment, and local residual enhancement is further introduced to preserve detailed responses after alignment:

(7) $${\tilde{X}}_{l}=AdaMaxPool(X_{l},S)+AdaAvgPool(X_{l},S)$$

(8) $$X_{l}^{\prime }={\tilde{X}}_{l}+\left({\tilde{X}}_{l}-{AvgPool}_{3\times 3}({\tilde{X}}_{l})\right)$$

For the low-resolution branch, due to its lower spatial resolution, nearest-neighbor interpolation is first employed to recover the target scale, while local average responses are incorporated to compensate for contextual information during scale restoration:

(9) $${\tilde{X}}_{s}={\mathrm{Upsample}}_{nearest}(X_{s},S)$$

(10) $$X_{s}^{\prime }={\tilde{X}}_{s}+{\mathrm{AvgPool}}_{3\times 3}({\tilde{X}}_{s})$$

For the intermediate-resolution branch, since its scale is already consistent with the target size, the original feature $X_{m}$ is retained directly. The aligned features from the three branches are subsequently merged along the channel dimension:

(11) $$Y=\mathrm{Concat}(X_{l}^{\prime },X_{m},X_{s}^{\prime })$$

**Figure 6:**
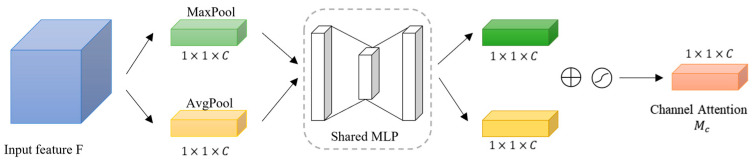
Architecture of the CA module.

To further improve feature integration after multi-scale fusion, a 1×1 convolution is introduced for channel reorganization and linear projection. This operation enables effective interaction among features from different levels while maintaining the spatial resolution of the feature map, thus producing a more compact fused representation. On this basis, a channel attention (CA) module is further incorporated to adaptively recalibrate the fused feature [17], as shown in Figure 6. Specifically, the module extracts channel descriptors by means of global average pooling and global max pooling, models inter-channel dependencies using a shared transformation branch, and generates corresponding channel weights to reweight the input features. With this mechanism, the network can enhance informative responses and suppress redundant background interference, thereby improving feature quality and detection performance.

In summary, MACFPN aligns and fuses features at different scales, allowing the fine-grained localization information of small objects and high-level semantic information to jointly participate in detection, thereby improving detection accuracy. Meanwhile, its scale transformation mainly relies on parameter-free pooling and upsampling operations, and a 1×1 convolution is used to compress redundant channels, enabling multi-scale feature fusion to be achieved with relatively low computational cost.

#### 2.2.3. NWDR Loss

Traditional IoU-based bounding-box regression losses are sensitive to slight positional offsets and scale variations in small-object detection. When the target box is small, minor center-point offsets or width–height variations can cause large fluctuations in the IoU value, thereby affecting localization accuracy and training stability. Therefore, this study proposes NWDR Loss. While preserving the geometric constraints of CIoU, NWDR Loss introduces the normalized Wasserstein distance and combines it with a dynamic weighting mechanism to reweight the CIoU regression term, thereby enhancing regression supervision for small-object samples [18].

Let the predicted box and the ground-truth box be defined as

(12) $$B_{p}=(c_{x}^{p},c_{y}^{p},w^{p},h^{p}),B_{g}=(c_{x}^{g},c_{y}^{g},w^{g},h^{g})$$

where $c_{x}$ and $c_{y}$ represent the center coordinates, whereas w and h denote the width and height, respectively. Unlike IoU, which relies only on the overlapping region, the Wasserstein distance considers both center position and scale differences, making it more suitable for describing the geometric deviation between small object boxes. The squared Wasserstein distance is defined as

(13) $$W^{2}(B_{p},B_{g})=(c_{x}^{p}-c_{x}^{g}{)}^{2}+(c_{y}^{p}-c_{y}^{g}{)}^{2}+\frac{\left(w^{p}-w^{g}{)}^{2}\right.}{4}+\frac{\left(h^{p}-h^{g}{)}^{2}\right.}{4}$$

To reduce the influence of target scale on distance measurement, the normalization coefficient for the i-th positive sample is defined as

(14) $$c_{i}=max(\beta \sqrt{\left(w_{i}^{g}{)}^{2}+(h_{i}^{g}{)}^{2}+\varepsilon \right.},\;1)$$

where β is the scale adjustment coefficient used to control the influence of the ground-truth box size on the normalization coefficient $c_{i}$, and ε is a small positive constant used to avoid numerical instability in square-root and division operations, as well as in subsequent related calculations. Through the max(⋅,1) operation, $c_{i}$ is constrained to be no smaller than 1.

The normalized Wasserstein distance is expressed as

(15) $$NWD_{i}=exp(\;-\frac{W_{i}^{2}+\varepsilon }{c_{i}})$$

To increase the regression weight of small-object samples, the dynamic enhancement factor is defined as

(16) $$s_{i}=min(max(1+\frac{1}{\sqrt{w_{i}^{g}h_{i}^{g}+\varepsilon }}-1,\;1),\;3)$$

where $s_{i}$ adjusts the sample weight according to the ground-truth box area. The smaller the target scale, the larger the value of $s_{i}$. Its maximum value is set to 3 to avoid excessive enhancement weights for extremely small objects, which could affect training stability.

Based on this, the dynamic weight is formulated as

(17) $$\lambda_{i}=1+s_{i}(1-NWD_{i}{)}^{2}$$

The dynamic weight $\lambda_{i}$ is used to reweight the CIoU regression term for each positive sample, thereby enhancing the regression supervision for small objects and difficult-to-localize samples.

The final NWDR Loss is formulated as

(18) $$L_{box}=\frac{1}{N_{pos}}\sum_{i=1}^{N_{pos}}\lambda_{i}(1-CIoU_{i})$$

where $N_{pos}$ denotes the total number of positive samples. Overall, NWDR Loss combines CIoU, normalized Wasserstein distance, and dynamic weighting to improve the stability and focus of bounding-box regression for small objects.

#### 2.2.4. Development of Software System

**Figure 7:**
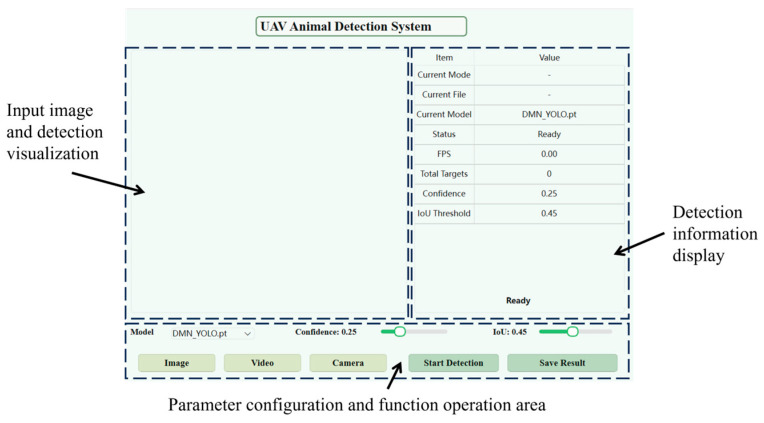
UAV-based Grazing Animal Detection System Interface.

To meet the functional requirements of image input, target recognition, result visualization, and parameter configuration in UAV-based animal detection scenarios, the software interface was designed and implemented based on PySide6 6.7.2. The system is capable of recognizing animal targets in the input images and providing real-time feedback on the current mode, file name, model information, running status, detection frame rate, number of targets, and related threshold parameters. In addition, it supports model switching, parameter adjustment, detection initiation, and result saving, and provides multiple input modes, including image, video, and camera. The system interface is shown in Figure 7.

## 3. Results

### 3.1. Experimental Environment

All experiments in this study were implemented based on the PyTorch deep learning framework. The experimental setup included Python 3.11.11, PyTorch 2.2.2, and CUDA 12.1. Model training and inference were performed on a computing platform configured with an NVIDIA GeForce RTX 4090 (24 GB) GPU. The primary training settings were as follows: the input images were uniformly resized to 640 × 640 pixels, the training was conducted for 100 epochs, and the batch size was fixed at 64. The SGD optimizer was employed, with an initial learning rate of 0.01 and a decay coefficient of 0.01, resulting in a final learning rate of 0.0001. The momentum coefficient and weight decay were set to 0.937 and 0.0005, respectively.

To assess the effectiveness of the proposed method, evaluation indicators were selected from two aspects, namely recognition accuracy and computational efficiency. Recognition accuracy was evaluated using Precision (P), Recall (R), F1-score, and mean Average Precision (mAP). The expressions for Precision and Recall are given below:

(19) $$P=\frac{TP}{TP+FP}$$

(20) $$R=\frac{TP}{TP+FN}$$

where TP, FP, and FN represent true positives, false positives, and false negatives, respectively. TP denotes the number of targets correctly identified by the model; FP denotes the number of background objects or objects from other categories that are incorrectly identified as the current category; and FN denotes the number of targets from the current category that are missed or incorrectly identified as other categories. Precision reflects the correctness of the model’s prediction results, while Recall reflects the model’s ability to detect ground-truth targets.

The F1-score was used to jointly assess Precision and Recall, and it is calculated as follows:

(21) $$F1=\frac{2PR}{P+R}$$

Average Precision (AP) refers to the area under the precision-recall curve for a given class, whereas mAP denotes the average AP across all classes, which can be written as

(22) $$mAP=\frac{1}{N}\sum_{i=1}^{N}AP_{i}$$

where N denotes the number of target classes used for mAP calculation, and $AP_{i}$ denotes the average precision of the i-th class. In this study, cattle, sheep, and wild animals were selected as the detection targets; therefore, N was set to 3. The mAP was obtained by averaging the AP values of these three classes. In this study, mAP@0.5 and mAP@0.5:0.95 were used as the detection accuracy evaluation metrics. Specifically, mAP@0.5 refers to the mean Average Precision at an Intersection over Union (IoU) threshold of 0.5, while mAP@0.5:0.95 refers to the mean Average Precision averaged over IoU thresholds from 0.5 to 0.95 with a step size of 0.05.

Model efficiency was evaluated in terms of parameter count (Parameters), floating-point operations (FLOPs), and model size (Model Size).

### 3.2. Experimental Results and Analysis

#### 3.2.1. Comparison of Downsampling Modules

To evaluate the effectiveness of the DSDown downsampling module, comparison experiments were carried out with several commonly used lightweight or downsampling structures, including GhostConv [19], DWConv, DynamicConv [20], and ADown [21]. These modules are all lightweight feature extraction or downsampling structures commonly used to reduce model complexity and computational cost. The results are summarized in Table 2.

As shown in Table 2, DSDown exhibited strong overall performance under all evaluation metrics, achieving a precision of 92.9%, a recall of 89.4%, and an mAP@0.5 of 95.1%. Among all compared methods, DSDown obtained the highest recall and mAP@0.5, indicating that it has a stronger capability for perceiving and representing small targets in complex grassland backgrounds. Compared with GhostConv and DWConv, DSDown improved precision, recall, and mAP@0.5 to varying degrees, suggesting that conventional lightweight convolutions alone are insufficient to balance detail preservation and semantic representation during downsampling, whereas DSDown, through the collaborative modeling of the semantic branch and the detail branch, can more effectively enhance target-related features for small-object detection. Compared with DynamicConv, although the precision of DSDown was slightly lower by 0.2 percentage points, whereas its recall and mAP@0.5 increased by 0.8 and 1.1 percentage points, respectively, while the number of parameters was significantly reduced from 4.04 M to 2.33 M, indicating that DSDown achieves a better balance between detection performance and model complexity. Compared with ADown, DSDown improved recall and mAP@0.5 by 2.0 and 1.0 percentage points, respectively. Although its precision was 0.1 percentage points lower, while its parameter count and FLOPs increased by 0.10 M and 0.4 G, respectively, its overall detection performance remained superior, demonstrating that the proposed dual-branch downsampling structure can more effectively preserve the edge, texture, and local structural information of small targets at an acceptable computational cost, thereby improving detection capability in complex grassland scenes. Overall, DSDown achieves better comprehensive detection performance while maintaining lightweight characteristics, indicating stronger suitability for UAV-based small-object detection in grassland scenarios.

#### 3.2.2. Sensitivity Analysis of the β Parameter in NWDR Loss

To further analyze the influence of the scale normalization coefficient β in NWDR Loss on detection performance, a parameter sensitivity experiment was conducted. In NWDR Loss, β is used to adjust the normalization scale of the Wasserstein-distance term, thereby controlling the sensitivity of the NWD-based dynamic weighting to geometric deviations between predicted boxes and ground-truth boxes.

**Figure 8:**
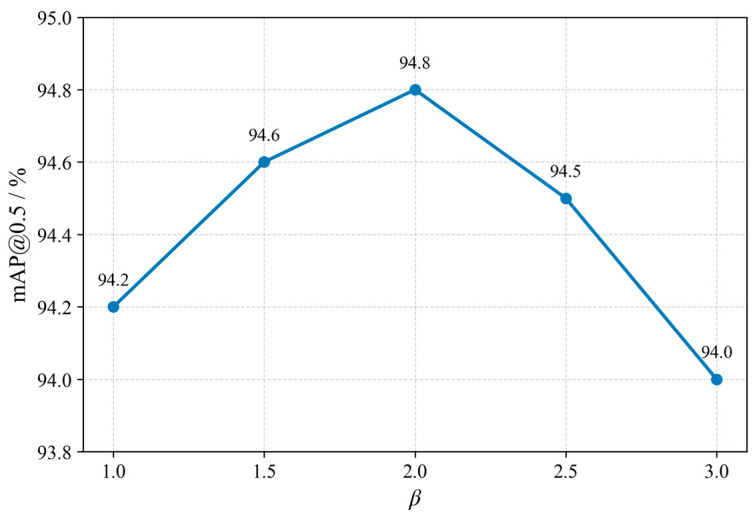
Effect of the β parameter in NWDR Loss on mAP@0.5.

In this experiment, β was set to 1.0, 1.5, 2.0, 2.5, and 3.0, respectively, while all other training settings were kept unchanged. Only mAP@0.5 was used for comparison to evaluate the influence of different β values on the detection performance. The experimental results are shown in Figure 8.

As shown in Figure 8, the mAP@0.5 first increased and then decreased as β increased. When β increased from 1.0 to 2.0, the mAP@0.5 improved from 94.2% to 94.8%, indicating that an appropriate increase in β can strengthen the regression supervision of small-object samples and help the model better learn the localization information of small-scale animal targets in UAV images. When β was set to 2.0, the model achieved the highest mAP@0.5 of 94.8%, demonstrating that this setting provides a good balance between enhancing small-object localization and maintaining training stability.

However, when β was further increased to 2.5 and 3.0, the mAP@0.5 decreased to 94.5% and 94.0%, respectively. This indicates that an excessively large β may overly smooth the Wasserstein-distance-based modulation, reducing the sensitivity of the dynamic weight to geometric deviations between predicted boxes and ground-truth boxes. As a result, the regression supervision for hard-to-localize small targets may be weakened, thereby limiting further improvement in detection performance. In UAV-based grassland scenes involving multiple animal categories and target scales, an inappropriate β value may disturb the optimization balance between regression sensitivity and training stability.

Therefore, considering both small-object detection performance and training stability, β was set to 2.0 in NWDR Loss and used as the default parameter in the subsequent experiments.

#### 3.2.3. Effect of Different Loss Functions on Detection Performance

To further evaluate the influence of NWDR Loss on detection performance, comparative experiments were performed using several widely adopted bounding-box regression loss functions, including CIoU, EIoU [22], DIoU [23], SIoU [24], and GIoU [25]. The comparison results are shown in Table 3.

As shown in Table 3, different bounding-box regression loss functions had varying effects on detection performance. When CIoU was used as the baseline, the model showed relatively stable overall performance. EIoU showed the weakest performance in recall, mAP@0.5, and mAP@0.5:0.95, indicating limited suitability for small-object localization in the current task. DIoU improved over EIoU, but its overall performance was still inferior to that of the other loss functions. SIoU achieved the highest precision of 93.1%, showing an advantage in classification discrimination, whereas its recall and mAP@0.5:0.95 were not optimal. GIoU produced relatively balanced results, but its overall performance still remained below that of NWDR Loss. In contrast, the proposed NWDR Loss achieved the best recall and mAP@0.5, reaching 89.3% and 94.8%, respectively, while maintaining a high precision of 92.7%. Relative to the baseline CIoU, NWDR Loss improved precision, recall, and mAP@0.5 by 0.3, 0.1, and 0.3 percentage points, respectively. These findings suggest that NWDR Loss can further improve the model’s sensitivity to small-object bounding-box regression and enhance overall detection performance.

#### 3.2.4. Ablation Experiment

As shown in Table 4, the three proposed improvements all contributed positively to the baseline model from different aspects. After introducing DSDown alone, the precision, recall, mAP@0.5, and mAP@0.5:0.95 of the model increased by 0.5, 0.2, 0.6, and 1.0 percentage points, respectively. This indicates that, through the collaborative modeling of the semantic branch and the detail branch, DSDown can effectively preserve the edge, texture, and local structural information of small targets while reducing spatial resolution and computational cost. After introducing MACFPN, the precision, recall, mAP@0.5, and mAP@0.5:0.95 increased by 0.8, 0.3, 0.8, and 0.9 percentage points, respectively, suggesting that this structure can more sufficiently reorganize shallow detail information, intermediate structural information, and deep semantic information through cross-layer scale alignment and multi-branch fusion. When NWDR Loss was adopted, the mAP@0.5 increased by 0.3 percentage points, whereas mAP@0.5:0.95 showed slight fluctuations. This suggests that, after introducing the normalized Wasserstein distance and dynamic weighting mechanism, the model can more accurately characterize the distribution differences between the predicted box and the ground-truth box in terms of position and scale, thereby improving localization accuracy and training stability. In particular, when DSDown and MACFPN were used together, they formed a favorable complementary relationship between feature fidelity and cross-scale fusion. This allowed the contour and positional information of small targets to be more sufficiently preserved during feature fusion, thereby enhancing the completeness and discriminability of small-target feature representations and increasing the model’s mAP@0.5:0.95 to 58.4%. Similarly, when DSDown was combined with NWDR Loss, the enhanced feature preservation during downsampling and the optimized regression constraints jointly improved the model’s detection performance, indicating that more informative feature representations can be further converted into localization gains under more effective bounding-box supervision. When MACFPN and NWDR Loss were combined, the enhanced feature representation capability and optimized bounding-box regression constraints produced a synergistic effect, increasing mAP@0.5 to 95.5% and mAP@0.5:0.95 to 57.4%, while reducing the parameter count to 1.91 M. Finally, when all three improvements were incorporated, the model achieved collaborative optimization in feature downsampling, cross-layer fusion, and bounding-box regression. The precision, recall, and mAP@0.5 reached 93.6%, 89.9%, and 95.8%, respectively. The mAP@0.5:0.95 of the complete model was 58.2%, slightly lower than the 58.4% obtained without NWDR Loss. This may be because the stronger regression constraints introduced by NWDR Loss increase the optimization influence of small-object samples in terms of center-position and scale differences, thereby altering the original optimization balance for fine-grained bounding-box fitting to some extent. Since mAP@0.5:0.95 is more sensitive to bounding-box overlap under high IoU thresholds, this metric showed a slight decrease after introducing NWDR Loss. Overall, the mAP@0.5:0.95 of the complete model was still improved by 1.7 percentage points compared with the baseline model. Meanwhile, the parameter count, FLOPs, and model size were reduced by 35.7%, 14.3%, and 29.3%, respectively, indicating that the three improvements still show good complementarity and synergistic gains in terms of overall detection performance and lightweight design.

**Figure 9:**
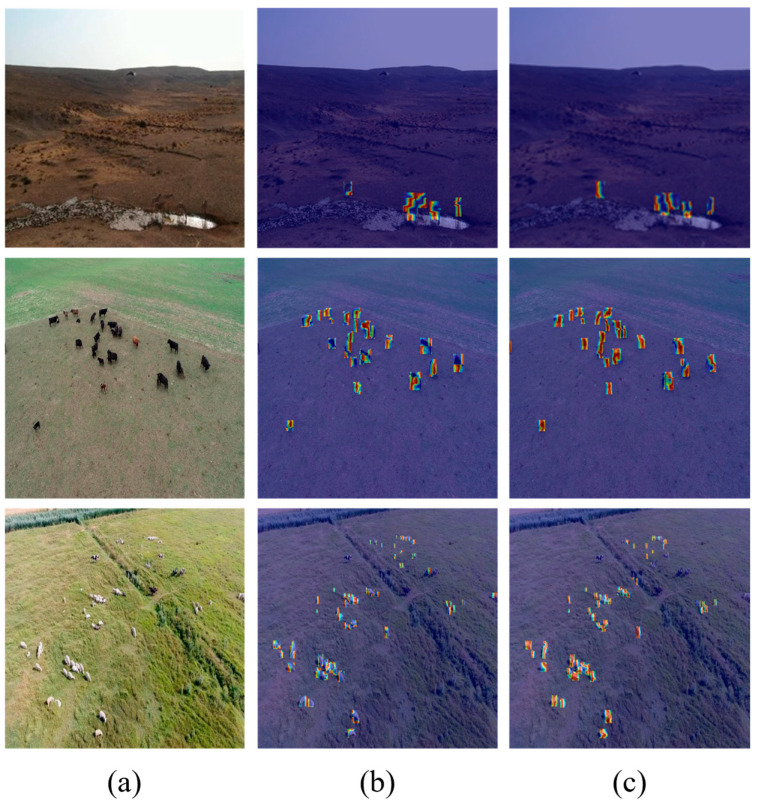
Grad-CAM heatmap comparison between YOLO11n and DMN-YOLO: (**a**) original UAV images; (**b**) Grad-CAM heatmaps generated by YOLO11n; (**c**) Grad-CAM heatmaps generated by DMN-YOLO.

Figure 9 presents a comparison of the Grad-CAM heatmaps [26] between the baseline YOLO11n model and the improved DMN-YOLO model in the UAV-based animal detection task. Compared with YOLO11n, DMN-YOLO exhibits denser and more continuous high-response regions over the target areas, enabling more accurate attention to animal locations while effectively suppressing irrelevant interference from complex grassland backgrounds. In particular, under scenarios involving small targets, densely distributed targets, and complex background textures, the improved model still maintains strong responses to the target regions and demonstrates better feature focusing ability.

#### 3.2.5. Comparative Experiment

To comprehensively validate the detection performance of the proposed DMN-YOLO in UAV-based grazing monitoring scenarios, this study selected a variety of mainstream object detection models for comparative experiments, the comparison results are shown in Table 5.

Compared with Faster-RCNN, DMN-YOLO improved precision, recall, and mAP@0.5 by 45.4, 10.6, and 23.5 percentage points, respectively, while significantly reducing the number of parameters, FLOPs, and model size. 

Compared with lightweight YOLO-series models, including YOLO11n [14], YOLOv9t [21], YOLOv8n [27], YOLOv5n [28], YOLOv10n [29], YOLO12n [30], and YOLO26n [31], and YOLO26n [31], DMN-YOLO achieved better overall performance in recall and mAP@0.5. Except for YOLOv9t, which showed slightly higher precision than DMN-YOLO, DMN-YOLO achieved varying degrees of improvement in most accuracy metrics. In particular, in terms of mAP@0.5, DMN-YOLO outperformed the above models by 2.3, 1.4, 0.9, 2.0, 1.3, 2.4, and 3.5 percentage points, respectively.

Compared with RT-DETR-R18 [32], DMN-YOLO improved precision, recall, and mAP@0.5 by 12.6, 10.3, and 10.2 percentage points, respectively. Meanwhile, its parameters, FLOPs, and model size were reduced from 19.8 M, 57.0 G, and 38.7 MB to 1.7 M, 5.4 G, and 3.7 MB, respectively, demonstrating superior detection accuracy and lightweight performance.

Compared with Drone-YOLO [33], DMN-YOLO improved precision, recall, and mAP@0.5 by 1.0, 1.0, and 1.6 percentage points, respectively, while reducing the parameter count and FLOPs from 2.9 M and 12.4 G to 1.7 M and 5.4 G. Overall, DMN-YOLO achieved a good balance between detection accuracy and lightweight design, with precision, recall, and mAP@0.5 reaching 93.6%, 89.9%, and 95.8%, respectively, while the number of parameters, FLOPs, and model size were only 1.7 M, 5.4 G, and 3.7 MB, further demonstrating its practical potential in resource-constrained UAV-based grazing monitoring scenarios.

**Figure 10:**
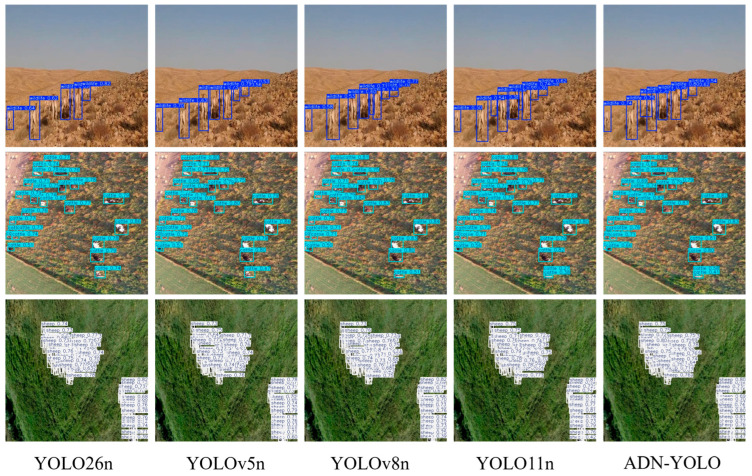
Detection results of different models.

Figure 10 shows the detection results of different models in typical UAV-based grassland monitoring scenarios. It can be observed that the models are able to detect animal targets under different shooting heights, target densities, and background conditions, while noticeable differences remain in challenging cases. In scenes with dense distribution, small targets, or complex grassland textures, the proposed DMN-YOLO produces more complete detection boxes and clearer category labels, and shows better robustness against missed detections and false detections. Even when the targets are sparsely distributed, partially occluded, or located far from the imaging center, the proposed model can still maintain reliable detection performance.

#### 3.2.6. Edge Device Deployment Experiment

**Figure 11:**
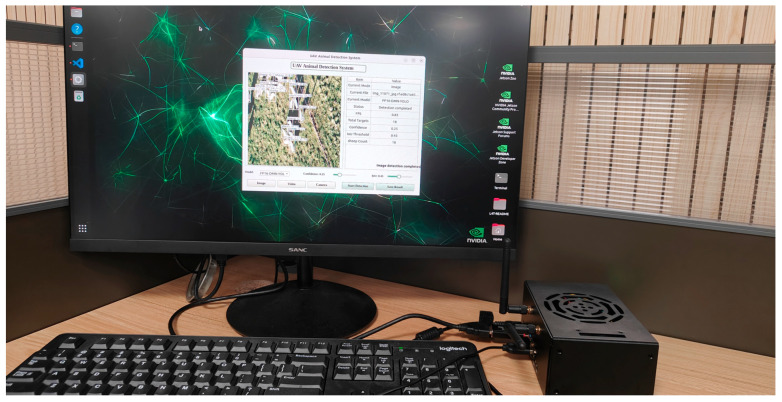
Edge-side deployment interface of the UAV-based grazing animal detection system.

To further validate the practical deployability of the proposed DMN-YOLO model on resource-constrained edge devices and meet the real-time detection requirements of UAV-based grassland grazing monitoring scenarios, edge-side deployment experiments were conducted on an NVIDIA Jetson Nano (NVIDIA Corporation, Santa Clara, CA, USA) embedded platform. The Jetson Nano has low power consumption and relatively limited computational resources, and can represent a typical lightweight edge-computing terminal. During deployment, the trained DMN-YOLO model weights were first converted into ONNX format, and then a TensorRT inference engine was constructed. FP16 half-precision inference and INT8 integer quantization inference were further adopted to accelerate the model, so as to analyze the influence of different low-precision deployment strategies on detection accuracy and inference efficiency. The edge-side deployment interface of the UAV-based grazing animal detection system is shown in Figure 11.

After adopting FP16 half-precision inference, DMN-YOLO achieved 93.22% mAP@0.5 and 56.17% mAP@0.5:0.95 on the Jetson Nano platform, with an inference speed of 57.76 FPS. Although this speed was slightly lower than the 60.56 FPS achieved by YOLO11n on the same platform, the decrease was only 2.80 FPS, indicating a limited reduction. Meanwhile, DMN-YOLO showed better overall performance in terms of detection accuracy, parameter count, computational cost, and model size. After adopting INT8 integer quantization inference, the inference speed of DMN-YOLO was further increased to 66.45 FPS, demonstrating stronger real-time inference capability.

In summary, after TensorRT-based low-precision acceleration, DMN-YOLO can achieve real-time inference on the resource-constrained Jetson Nano platform. These results demonstrate that the proposed model has good practical deployment potential in edge-side applications such as UAV-assisted grazing monitoring, grassland patrol, and intelligent pastoral-area management.

## 4. Discussion

The effectiveness of DMN-YOLO mainly arises from the synergistic effect of DSDown, MACFPN, and NWDR Loss. The proposed improvements enhance detection performance without increasing the burden of edge-side deployment. After introducing DSDown, the model achieved 92.9% precision, 89.4% recall, 95.1% mAP@0.5, and 57.5% mAP@0.5:0.95, demonstrating the best overall performance among GhostConv, DWConv, DynamicConv, and ADown. After introducing MACFPN, the number of parameters was further reduced to 1.91 M. Meanwhile, NWDR Loss is only used during the training stage and does not introduce additional parameters or computational cost during inference. Compared with YOLO11n, the complete DMN-YOLO reduces the number of parameters, FLOPs, and model size by 35.7%, 14.3%, and 29.3%, respectively, indicating that the model maintains good lightweight characteristics and has potential for edge-side deployment. Furthermore, the complete model achieves 93.6% precision, 89.9% recall, 95.8% mAP@0.5, and 58.2% mAP@0.5:0.95, all of which are improved to varying degrees compared with the baseline YOLO11n. These results demonstrate that the proposed method can effectively enhance the detection capability for small-scale animal targets in UAV-based grassland scenes.

Although the proposed method achieves good detection performance, it still has certain limitations in practical UAV-based grassland monitoring scenarios. First, the model performance may be affected by target visibility and imaging conditions. When the color or texture of animals is highly similar to the background, such as yellow cattle in dry grass, the edge and texture differences between the foreground and background become significantly weaker. In this case, even though DSDown can preserve more local details, it may still be difficult to sufficiently distinguish target responses from background interference. When animal targets are extremely small, heavily occluded, or surrounded by dense grassland textures, MACFPN may introduce certain background noise while enhancing cross-scale information interaction. In addition, NWDR Loss mainly improves the stability of bounding-box localization, but it cannot directly solve classification confusion caused by similar appearances or weak foreground–background contrast. Meanwhile, as the UAV flight height increases, animal targets occupy fewer pixels in the image, and their contour and texture information become weaker. Strong illumination, shadows, low image sharpness, and motion blur may also lead to missed detections or localization errors. Second, since DMN-YOLO is trained based on predefined animal categories, it may still have limited generalization ability when new animal species that are not included in the training set appear in practical monitoring scenarios. Unseen species with appearances similar to known categories may be misclassified as existing categories, whereas species with larger differences in shape, color, or texture may be missed. Therefore, future work should further evaluate the robustness of the model under more diverse flight heights, imaging conditions, and open-category scenarios. Expanding animal categories, increasing complex environmental samples, and introducing open-set detection or incremental learning strategies may further improve the generalization ability of the model.

In addition, based on the above image-level detection capability, DMN-YOLO can also provide a foundation for video analysis tasks in practical grazing monitoring. The category labels and bounding-box locations output by the model can serve as front-end detection results for multi-object tracking algorithms, enabling target association between adjacent video frames and further supporting animal number estimation and dynamic distribution analysis. Future work will combine multi-object tracking algorithms with temporal information from UAV videos to extend the proposed method from single-frame image detection to tracking-assisted counting, thereby improving its application value in intelligent grazing monitoring and grassland ecological patrol.

## 5. Conclusions

This work presents DMN-YOLO, a lightweight detection framework for UAV-based monitoring of livestock and wild herbivores in complex grassland environments. Built upon YOLO11n, the model integrates three targeted improvements, namely the DSDown lightweight downsampling module, the MACFPN cross-scale feature fusion structure, and NWDR Loss for small-object bounding-box regression. These designs effectively address key challenges in grassland aerial imagery, including small target scales, obvious scale variations, complex backgrounds, and the demand for edge-side deployment.

Overall, this method provides a practical technical solution for the synchronous detection and dynamic monitoring of livestock and wild herbivores in open grassland environments, showing promising application potential in grazing management, ecological patrol, and smart pastoral-area monitoring. In future work, this framework can be further extended by integrating multi-object tracking and counting strategies, with dedicated counting metrics introduced to evaluate animal number estimation under dense distribution, occlusion, and varying illumination conditions.

## Figures and Tables

**Table 1:** Class distribution of annotation boxes in the constructed dataset.

Dataset Split	Number of Images	Wildlife	Cattle	Sheep
Training set	4613	3239	30,335	62,504
Validation set	1312	839	8666	17,893
Test set	702	490	4568	9856

**Table 2:** Comparison among different downsampling modules.

Model	P/%	R/%	mAP@0.5/%	mAP@0.5:0.95/%	Params/M	FLOPs/G
GhostConv	92.5	87.4	93.5	55.5	2.35	5.8
DWConv	91.9	87.1	93.2	54.7	2.10	4.9
DynamicConv	93.1	88.6	94.0	56.7	4.04	4.9
ADown	93.0	87.4	94.1	56.0	2.23	5.3
DSDown	92.9	89.4	95.1	57.5	2.33	5.7

**Table 3:** Comparison among Different Loss Functions.

Loss	P/%	R/%	mAP@0.5/%	mAP@0.5:0.95/%	F1/%
CIoU	92.4	89.2	94.5	56.5	90.8
EIoU	92.2	87.1	93.3	55.9	89.6
DIoU	92.1	88.1	94.0	56.2	90.1
SIoU	93.1	89.1	94.5	56.1	91.1
GIoU	92.2	88.4	94.3	56.4	90.3
NWDR Loss	92.7	89.3	94.8	56.3	91.0

**Table 4:** Ablation Experiment Results. “√” indicates that the corresponding module is adopted, while “×” indicates that the module is not used.

DSDown	MACFPN	NWDR Loss	P/%	R/%	mAP@0.5/%	mAP@0.5:0.95/%	Params/M	FLOPs/G	Model Size/MB
×	×	×	92.4	89.2	94.5	56.5	2.58	6.3	5.23
√	×	×	92.9	89.4	95.1	57.5	2.33	5.7	5.0
×	√	×	93.2	89.5	95.3	57.4	1.91	6.0	4.1
×	×	√	92.7	89.3	94.8	56.3	2.58	6.3	5.23
√	√	×	93.4	89.7	95.6	58.4	1.66	5.4	3.7
√	×	√	93.1	89.5	95.3	57.2	2.33	5.7	5.0
×	√	√	93.3	89.6	95.5	57.4	1.91	6.0	4.1
√	√	√	93.6	89.9	95.8	58.2	1.66	5.4	3.7

**Table 5:** Comparison of Different Models.

Model	P/%	R/%	mAP@0.5/%	Params/M	FLOPs/G	Model Size/MB
Faster-RCNN	48.2	79.3	72.3	41.1	180.3	244
YOLOv5n	92.3	88.3	93.5	2.5	7.1	5.0
YOLOv8n	93.6	89.6	94.4	3.0	8.1	6.0
YOLOv9t	94.4	89.7	94.9	2.0	7.6	4.7
YOLOv10n	91.5	87.8	93.8	2.3	6.5	5.6
YOLO11n	92.4	89.2	94.5	2.6	6.3	5.2
YOLO12n	91.4	88.0	93.4	2.6	6.3	5.3
YOLO26n	90.5	86.3	92.3	2.5	5.8	5.2
RT-DETR-R18	81.0	79.6	85.6	19.8	57.0	38.7
Drone-YOLO	92.6	88.9	94.2	2.9	12.4	6.4
DMN-YOLO	93.6	89.9	95.8	1.7	5.4	3.7

## Data Availability

The dataset utilized in this study is publicly available and sourced from the WAID dataset. The official dataset repository is available at https://github.com/xiaohuicui/WAID (accessed on 16 February 2026).
